# Secure communication through reliable S-box design: A proposed approach using coset graphs and matrix operations

**DOI:** 10.1016/j.heliyon.2023.e15902

**Published:** 2023-05-02

**Authors:** Abdul Razaq, Ghaliah Alhamzi, Sajida Abbas, Musheer Ahmad, Asima Razzaque

**Affiliations:** a Department of Mathematics, Division of Science and Technology, University of Education, Lahore, 54770, Pakistan; b Department of Mathematics and Statistics, College of Science, Imam Mohammad Ibn Saud Islamic University (IMSIU), Riyadh, 11564, Saudi Arabia; c Department of Computer Engineering, Jamia Millia Islamia, New Delhi, 110025, India; d Department of Basic Sciences, Deanship of Preparatory Year, King Faisal University Al Ahsa, Al Hofuf, Saudi Arabia

**Keywords:** Coset graphs, Substitution-box, Block cipher, Image encryption

## Abstract

Protection of sensitive information has been always the major security concern since decades to withstand against illegitimate access and usage. Substitution-boxes (S-boxes) are vital components of any modern day cryptographic system that allows us to ensure its resistance to attacks. The prime problem with creating S-box is that we are generally unable to discover a consistent distribution among its numerous features to withstand diverse cryptanalysis attacks. The majority of S-boxes investigated in the literature has good cryptographic defenses against some attacks but are susceptible to others. Keeping these considerations in mind, this paper proposes a novel approach for S-box design based on a pair of coset graphs and a newly defined operation of row and column vectors on a square matrix. Several standard performance assessment criteria are used to evaluate the reliability of proposed approach, and the results demonstrate that the developed S-box satisfies all criterions for being robust for secure communication and encryption.

## 1 Introduction

### 1.1 Background

In this era of the 21st century, where technology has advanced to new heights, researchers face significant difficulty when it comes to secure communication. Online data transfer from one place to another is widely done through various communication channels that demand privacy and protection. This protection can be achieved through the use of cryptography [1,2], which is a technique for safeguarding data and communication by using codes so that only the intended recipient can understand it. In computer science, the term “cryptography” relates to safe communication and information methods that use mathematical ideas and a system of calculations to transform messages in such a way that they become difficult to decode [3]. These methods are employed in designing cryptographic keys, digital signatures, and confirmation to maintain online browsing over the internet and private communications, transactions with credit cards and email, and confidential information.

There are two major branches of cryptography: asymmetric key cryptography [4] and symmetric key cryptography [5]. Asymmetric key cryptography employs two keys: one public and one private. The sender is linked to the public key, which is utilized to encrypt messages. When the message is delivered, it can only be decoded using the private key possessed by the recipient. The present study deals with symmetric cryptography. There are two different approaches to using symmetric cryptographic techniques to encipher plaintext: block ciphers and stream ciphers. In a stream cipher, one plaintext symbol is directly transformed into an encrypted text symbol. On the other hand, a series of plaintext symbols are encrypted as one block using block cipher [6]. An example of a block cipher is simple substitution, while columnar transposition is a stream cipher. Block ciphers are the most common symmetric encryption techniques used today.

A substitution-box (S-box), a vital integrant of symmetric-key cryptography, is used to execute substitution. In block ciphers, it is widely used to ensure the confusion property described by C.E. Shannon for concealing the connection between the key and the encrypted data [7,8]. One of the most secure block ciphers that encrypts plaintext and enables secure transmission is the Advanced Encryption Standard (AES) [9]. With the assistance of the substitution-box, the first phase, often known as the substitution step, involves performing byte substitution. This emphasizes the significance of S-box in the encryption process. It is the sole nonlinear constituent, and its robustness determines the performance and security of block ciphers.

The chosen S-box must be strong and demonstrate resistance to any cryptanalytic attempt. In an encryption scheme, nonlinearity is regarded as the S-box's leading performance criterion. Researchers have been interested in obtaining S-boxes that are both algebraically and cryptographically resilient for more than a decade. These S-boxes display many striking characteristics and provide intriguing outcomes for various ciphers. However, the primary goal is to increase the nonlinearity score of the S-boxes [10,11]. Security experts have become interested in evolving strong S-boxes, especially of the order of 8×8, as a result of their successful use in the AES block cipher.

### 1.2 Literature review

To form secure S-boxes, numerous innovative methods and procedures have recently been developed. In Ref. [12], an innovative method for designing S-boxes is discussed. The authors used a pair of chaotic and fitness maps in the algorithm of S-box design. An efficient method for constructing robust S-boxes involving chaotic maps and symmetry groups is devised by Javed et al. in Ref. [13]. With the use of a chaotic system, an initial S-box is constructed and then the proposed S-box is generated through the action of $S_{256}$ on initial S-box. In Ref. [14] a certain type of group theoretic graphs and a bijective function were utilized to generate a new S-box. Several performance indicators confirmed the robustness of suggested S-box. Lambic [15] designed a novel discrete-space chaotic function based on standard multiplication and circular shift. This map was then used to create an S-box with excellent security features. Anees and Ahmed [16] designed a potent S-box by investigating the behavior of van der pol oscillator. Firstly, the author used a technique to obtain the iterative solution of chaotic map. Then the ceiling function is employed to those solutions to achieve the task. A systematic scheme to evolve a S-box with high nonlinearity value is given in Ref. [17]. The chaotic map iteration yields a 16×16 matrix on which the genetic algorithm is applied to obtain suggested S-box. Mahboob et al. presented a robust S-box based on the reciprocal of degree 5 equation [18]. A new approach using zig-zag transformation is investigated through different chaotic systems for enhancing the efficiency of substitution-box structures based on chaos is examined in Ref. [19]. It has been shown that features of input S-box structure can be improved by exchanging the elements of S-box as per the zig-zag traversal. In Ref. [20] Zhang et al. proposed a method of encrypting images using newly designed 8 × 8 S-box which was constructed with the help of a new fractional order logistic map that has larger key space, more parameters, better efficiency than conventional logistic map. Whereas, Quiroga et al. in Ref. [21] utilized an integer chaotic lag time series generated from extended and improved logistic map to develop a scheme for constructing 8 × 8 S-boxes suited for high-speed secure communication. In Ref. [22], Yousaf et al. employed an efficient concept of permutation group theory to improvise the cryptographic strength of seed S-boxes. The effectiveness of the approach is tested over several S-boxes schemes where it has been found that post action of the approach results a strong structure of S-box which sufficiently better than seed S-boxes. In Ref. [23], a simple method of S-box generation is suggested which was based on a cascade discrete-time logistic-Chebyshev function which was applied to build an efficient image cryptosystem. The current study demonstrates a novel method to generate S-boxes having decent cryptographic resilience.

### 1.3 Analysis of weaknesses in existing designs and proposed scheme for improved cryptographic security

Since the design of S-boxes is a crucial component in the development of secure cryptographic systems. To achieve this, numerous algebraic and chaotic systems-based techniques have been proposed within the literature. However, it is important to note that despite the advantageous features conferred by these approaches, potential weaknesses have been identified that require careful consideration. In particular, S-box designs based solely on algebraic structures are vulnerable to attacks targeting the inherent algebraic structure of such designs. There are various attacks that can be executed on S-boxes constructed using algebraic techniques. Linear and differential attacks [24,25] are two examples of such attacks, which are designed to exploit the linear and differential characteristics of the S-box, respectively. XL attacks [26], Gröbner basis attacks [27], SAT solver attacks [28], side-channel attacks [29], XSL attacks [30], and Interpolation attacks [31] are other examples of attacks that can be executed on S-boxes based on algebraic constructions.

Similarly, despite the widespread adoption of chaotic systems in substitution box designs, several weaknesses have been identified in the literature. One of the critical issues is the algorithmic evolution of control parameters, which can result in periodicity and non-randomness in chaotic sequences [32,33]. This problem can compromise the security of cryptographic systems, as attackers can exploit the predictable nature of the chaotic sequences to launch attacks. Another weakness is the non-uniform distribution of chaotic sequences, which can result in biases and patterns that can be exploited by attackers. Furthermore, the finite precision effect can cause errors and inaccuracies in chaotic sequences, reducing the randomness and security of cryptographic systems. The dynamical degradation of chaotic functions is another issue that can significantly affect the security of cryptographic systems. The continuous evolution of control parameters can lead to a loss of randomness . Additionally, the limited number of control parameters and frail chaos in some chaotic systems can restrict the amount of randomness generated, limiting their suitability for cryptographic applications. The insufficient quantity of randomness can result in weak cryptographic keys, making the system vulnerable to attacks.

Moreover, the intrinsic evolution of algebraic structures and their automorphism nature represent a significant issue that must be addressed in the design of cryptographic systems. The automorphism nature of algebraic structures can introduce symmetries and patterns that can be exploited by attackers, compromising the security of cryptographic systems. Thus, in light of the weaknesses identified in existing S-box designs, there is a need to developed more innovative schemes for secure communication.

Alternatively, to achieve a high degree of randomness, researchers recommend the use of true random sequences generated by naturally occurring processes. True random sequences are characterized by their unpredictability, irreversibility, and unreproducibility, making them highly resistant to attacks by adversaries. The unpredictability of true random sequences is attributed to their generation from physical processes that are inherently random. The irreversibility of true random sequences means that once a sequence has been generated, it cannot be recreated or predicted even if an adversary has knowledge of its internal structure and response history.

The focus of recent research has been on developing techniques for extracting true random numbers for cryptographic applications. The proposed technique involves the integration of group theory and graph theory. The integration of these two theories enables the extraction of true random numbers, which are then used to construct cryptographically robust substitution boxes.

The major contributions of this paper are as follows:

1. There exists limited research on S-box design based on a single graph in the literature. In order to introduce more randomness and complexity, we propose the development of two graphs based on concepts from group theory.
2. We introduce a new operation using row and column vectors over a square matrix to generate a new matrix. We utilize this operation to enhance the robustness of the initial S-box and generate the proposed S-box.
3. The strength of the proposed S-box is tested using various benchmark algebraic parameters. The outcomes obtained from these evaluation tools validate the reliability and robustness of the proposed S-box in preventing several attacks.
4. We demonstrate the effectiveness of the proposed S-box in image encryption applications.

Thus, our contributions provide a novel approach to S-box design and demonstrate the potential of the proposed S-box for improving the security of various cryptographic applications.

The remainder of the paper is divided into the following sections. We outline some preliminary concepts and the novel operation on square matrix needed to set up the proposed S-box in section 2. Section 3 contains comprehensive detail about pair of coset graphs whose sum of vertices is 256. The proposed S-box design approach is presented in Section 4. Some well-known security tests are performed in Section 5 to assess the strength of the presented S-box. Several statistical studies are carried out in Section 6 to determine the acceptability of proposed S-box for image encryption applications. The conclusion of this work is described in Section 7.

## 2 Preliminaries

In this section, we go through a few mathematical notions that are relevant to suggested S-box design strategy.

### 2.1 Coset graphs of modular group over prime field

**Fig. 1:**
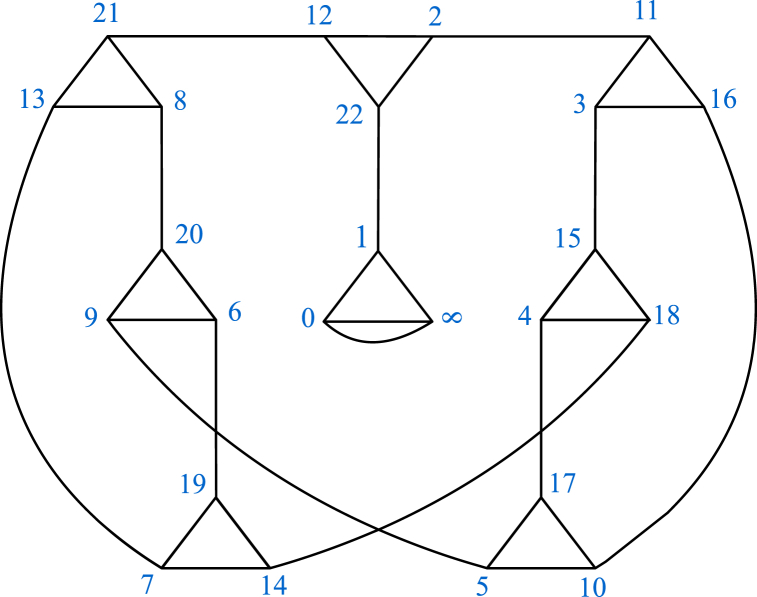
Coset graph of M over $F_{23}\cup \left\{\infty \right\}$.

The modular group *G* is a non-abelian group of infinite order generated by two elements α and β which are linear fractional transformations defined by α(x) = $\frac{-1}{x}$ and β(x) = $\frac{x-1}{x}$. The finite presentation of *G* is $\left\langle \alpha ,\beta =\alpha^{2}=\beta^{3}=1\right\rangle$. Due to its extensive applications across numerous scientific domains, the modular group *G* is considered as the most significant infinite discrete group. There is a long history of research into how *G* acts on various mathematical structure. Coset graphs for *G* over a prime field were established by Higman in the late 1970s. Since $\alpha^{2}=\beta^{3}=1$, therefore these graphs are made up of triangles which are joined together by edges. The edge of a triangle Δxyz (in anticlockwise order) linking vertices x and y is symbolized by β. A line, that is not an edge of a triangle, connecting two vertices (which may or may not of the same triangle) is denoted by the symbol α. It is important to mention that if the vertices that are joined by α lie on the same triangle, then a cap appears on such a triangle (See triangle Δ0.∞.1 in Fig. 1). We refer to Refs. [[34], [35], [36], [37]] for more information on coset graphs. It is well known that, for a prime number p, $F_{P}=\left\{0,1,2,\ldots ,p-1\right\}$ is a field. The action of *M* on $F_{p}\cup \left\{\infty \right\}$ leads to the formation of a finite coset graph. Since $\alpha \left(0\right)=\frac{-1}{0}$ = ∞, therefore, we take $F_{p}\cup \left\{\infty \right\}$ instead of simple field $F_{p}$. Let us draw a coset graph of *M* over $F_{23}\cup \left\{\infty \right\}=\left\{0,1,2,3,\ldots ,22,\infty \right\}$.

Following are the permutation forms of α and β determined by (α(x) = $\frac{-1}{x}$ and β(x) = $\frac{x-1}{x}$ respectively:

α:(0,∞)(1,22)(2,11)(3,15)(4,17)(5,9)(6,19)(7,13)(8,20)(10,16)(12,21)(14,18)

β:(0,∞,1)(2,12,22)(3,16,11)(4,18,15)(5,10,17)(6,20,9)(7,14,19)(8,21,13)

Since the permutation β is the product of 8 disjoint cycles of length 3, therefore the coset graph of $F_{23}\cup \left\{\infty \right\}$ has eight triangles. A triangle Δ9.6.20 (in anticlockwise order) is represented by the cycle (9,6,20). Similarly, all of the triangles are labelled. Furthermore, the permutation α is the product of 12 transpositions such that each of which connects any two vertices of the 8 triangles. For instance, the transposition (2,11) joins vertex 2 of Δ2.12.21 to vertex 11 of Δ11.3.16. In this way, the whole graph is emerged. Fig. 1 depicts the coset graph made up of the permutation representations of α and β.

### 2.2 Parametrization technique for evolving coset graphs

The coset graph shown in Fig. 1, is obtained from the natural action of *G* on $F_{23}\cup \left\{\infty \right\}$. All the vertices of such graphs are by fixed by $\alpha^{2},\beta^{3}$ and ${\left(\alpha \beta \right)}^{p}$. So, these graphs represent the diagrammatic version of the triangle group $\left\langle \alpha ,\beta =\alpha^{2}=\beta^{3}={\left(\alpha \beta \right)}^{p}=1\right\rangle$. In Ref. [38], it has been proved that, corresponding to each element *t* in $F_{p}$ a separate coset graph can be compose. The method that is used for drawing these types of graphs is called parametrization method. Irrespective of the natural action which allows only one coset graph, the parametrization process enables us to evolve *p* number of coset graphs for each $F_{p}$. Another advantage of parametrization method is that it allows us to generate coset diagrams over $F_{p}$ for several triangle groups $\left(2,3,n\right)=\left\langle \alpha ,\beta =\alpha^{2}=\beta^{3}={\left(\alpha \beta \right)}^{n}=1\right\rangle$, where n is divisor of p+1 or p−1. Next, we briefly illustrate the parametrization method.

Choose $F_{p}$, the prime field over which coset graph is intended to compose. Then select the divisor n of p+1 or p−1 so that diagrammatic form of $\left\langle \alpha ,\beta =\alpha^{2}=\beta^{3}={\left(\alpha \beta \right)}^{n}=1\right\rangle$ can be obtained. Each n is associated with a polynomial f(t) [38] whose solutions in $F_{p}$ provide a key to solve five equations of parametrization method (See Eqs 2.1, 2.5)). Let t be the one of the roots of f(t) and set α(x)=ax+kccx−a and β(x)=dx+kffx−d−1. Solve equations 2.1, 2.5 to find α and β.

2.1 t=r2Δ

2.2 $$r^{2}+ks^{2}-3=0$$

2.3 $$d^{2}+kd+f^{2}+1=0$$

2.4 a(2d+1)+2kcf−r=0

2.5 2af−c(2d+1)−s=0

The polynomials associated with first 20 values of n are written in Table 1. If one needs to obtain a polynomial for higher value of n, the method can be found in Ref. [38].

**Table 1:** Relationship between k and f(t).

f(t)	n
t−4	1
t	2
t−1	3
t−2	4
$t^{2}-3t+1$	5
t−3	6
$t^{3}-5t^{2}+6t-1$	7
$t^{2}-4t+2$	8
$t^{3}-6t^{2}+9t-1$	9
$t^{2}-5t+5$	10
$t^{5}-9t^{4}+28t^{3}-35t^{2}+15t-1$	11
$t^{2}-4t+1$	12
$t^{6}-11t^{5}+45t^{4}-84t^{3}+70t^{2}-21t+1$	13
$t^{6}-120t^{5}+55t^{4}-120t^{3}+126t^{2}-56t+7$	14
$t^{7}-13t^{6}+66t^{5}-165t^{4}+210t^{3}-126t^{2}+28t-1$	15
$t^{6}-12t^{5}+54t^{4}-112t^{3}+106t^{2}-40t+4$	16
${t^{8}-15t^{7}+91t}^{6}-286t^{5}+495t^{4}-462t^{3}+210t^{2}-36t+1$	17
$t^{6}-12t^{5}+54t^{4}-112t^{3}+105t^{2}-36t+3$	18
${t^{9}-17t^{8}+120t}^{7}-455t^{6}+1001t^{5}-1287t^{4}+924t^{3}-330t^{2}+45t-1$	19
${t^{8}-16t^{7}+104t}^{6}-352t^{5}+66t^{4}-680t^{3}+356t^{2}-80t+5$	20

We refer to Ref. [38] for a more detailed description and proof of this method.

### 2.3 A newly defined operation between row/column and square matrices

Here, we introduce a new operation between a row vector of order 1×n and a square matrix of order n.

Definition 2.1 Let A be a square matrix of order n with entries from $\left\{0,1,2,3,\ldots ,n^{2}-1\right\}$ and R be a row vector of n elements from {1,2,3,…,n}. Then the operation of R on A results a new square matrix $A^{\prime }$ of order n in the following manner.

t∈{1,2,3,…,n} is the ith element of R ⟺ tth row of $A^{\prime }$ is equal to ith row of A..

Similarly, we can define operation between a column vector C of order n×1 and an n×n matrix.

Example 2.1 Consider a 3×3 matrix $A=\left(\begin{array}{ccc} 6 & 2 & 4\\ 5 & 7 & 0\\ 1 & 3 & 8 \end{array}\right)$ over {0,1,2,3,4,5,6,7,8} and a row vector R=[231]. Then by Definition 2.1, the operation of R on A designs $A^{\prime }=\left(\begin{array}{ccc} 1 & 3 & 8\\ 6 & 2 & 4\\ 5 & 7 & 0 \end{array}\right)$.

## 3 Coset graphs employed in the proposed scheme

An 8×8 S-box has 256 elements and it is a 16×16 matrix with entries ranging from 0 to 255. Many researchers used the Galois field of order 256 in the construction of S-boxes. Some S-box construction methods involve the action of some suitable group on $F_{257}$. In this work, we have presented a new idea of evolving S-boxes involving a pair of prime fields, the sum of whose elements is 256. We find the following pairs of prime fields, $F_{i}$ and $F_{j}$ such as i+j=256.

i. $F_{251}$ and $F_{5}$ ii. $F_{227}$ and $F_{29}$ iii. $F_{239}$ and $F_{17}$ iv. $F_{197}$ and $F_{59}$.

v. $F_{233}$ and $F_{23}$ vi. $F_{173}$ and $F_{83}$ vii. $F_{167}$ and $F_{89}$ viii. $F_{107}$ and $F_{149}$.

We can take any of these pairs to form 8×8 S-box. In the present study, we use $F_{107}$ and $F_{149}$, as well as the parametrization technique to compose coset graphs for $\left\langle \alpha ,\beta =\alpha^{2}=\beta^{3}={\left(\alpha \beta \right)}^{18}=1\right\rangle$ and $\left\langle \alpha ,\beta =\alpha^{2}=\beta^{3}={\left(\alpha \beta \right)}^{4}=1\right\rangle$ over $F_{107}$ and $F_{149}$ respectively. This generates the initial S-box with decent cryptographic strength. To add more randomness and complexity, we use the row and column vectors on the initial seed S-box that leads to the suggested S-box

### 3.1 Implementation of parametrization techniques to compose coset graph over $F_{149}$

According to parametrization method, for each divisor n of 150 and 148, we can draw a coset graph for $\left\langle \alpha ,\beta =\alpha^{2}=\beta^{3}={\left(\alpha \beta \right)}^{n}=1\right\rangle$ over $F_{149}$. Here, we choose n=4 so that coset graph for $\left\langle \alpha ,\beta =\alpha^{2}=\beta^{3}={\left(\alpha \beta \right)}^{4}=1\right\rangle$ is composed. To find linear fractional transformations $\alpha \left(x\right)=\frac{ax+kc}{cx-a}$ and $\beta \left(x\right)=\frac{dx+kf}{fx-d-1}$ satisfying $\alpha^{2}=\beta^{3}={\left(\alpha \beta \right)}^{4}=1$, we need to solve Eqs 2.1, 2.5) for t=2. In Eq. (2.1), suppose Δ=2 to obtain r=2. Eq. (2.2) gives s=1 by assuming k=2. Take d=1 in Eq. (2.3), to get f=64. Substitute these values of r,k,s,d and f in Eqs. (2.4) and (2).5 and solve them to acquire a=12 and c=15. Ultimately, the linear fractional transformations $\alpha \left(x\right)=\frac{12x+30}{15x-12}$ and $\beta \left(x\right)=\frac{1x+128}{64x-2}$ are emerged which satisfy the relations $\alpha^{2}=\beta^{3}={\left(\alpha \beta \right)}^{4}=1$. Next, we apply α and β on all entries of $F_{149}$ to find out their permutation representations. Note that, for all $x\in F_{149}$*,* basically α(x)andβ(x) are non-integral (fractional) numbers. Since 149 ≡0, therefore add 149 to the numerator until the denominator divides it. For example: α(0) = $\frac{12\left(0\right)+30}{15\left(0\right)-12}$ = $\frac{30}{-12}$ = $\frac{30+\left(-6\right)\times 149}{-53}$ = $\frac{-864}{-12}$ = 72..

Similarly, we can find α(x) and β(x) for all $x\in F_{149}\cup \left\{\infty \right\}$ and write those in permutation format:

α=(72,0)(14,1)(3,2)(4,132)(44,5)(93,6)(7,131)(8,26)(52,9)(27,10)(11,115)(17,12)

(13,23)(15,129)(16,99)(18,46)(91,19)(122,20)(21,116)(22,119)(24,60)(28,25)

(29,96)(56,30)(67,31)(41,32)(33,136)(34,105)(35,61)(36,94)(37,126)(38,97)

(40,48)(92,42)(43,51)(45,73)(47,86)(49,148)(59,50)(53,143)(54,114)(146,55)

(135,57)(104,58)(62,144)(63,66)(64,81)(65,83)(68,78)(69,121)(70,124)(118,71)

(74,79)(141,75)(76,111)(77,90)(125,80)(84,109)(85,147)(87,108)(88,89)(95,128)

(98,102)(100,130)(101,107)(103,123)(106,113)(110,140)(112,145)(117,137)

(120,∞)(127,134)(133,139)(138,142)

β=(21,0,85)(14,∞,7)(1,91,47)(38,36,147)(143,2,98)(76,148,50)(3,25,138)(95,137,49)

(80,4,146)(100,74,108)(5,29,113)(58,77,89)(6,69,63)(52,41,99)(114,8,40)(67,37,115)(64,9,136)(46,66,135)(97,10,131)(116,119,86)(39,11,73)(51,54,84)(34,12,106)(104,124,35)(56,130,13)(103,55,133)(101,15,107)(71,129,118)

(16,57,141)(112,81,93)(90,24,17)(70,62,96)(18,32,145)(75,121,33)(72,19,27)

(22,94,120)(79,20,123)(23,134,132)(82,26,65)(110,126,109)(28,78,111)(102,42,117)(122,30,125)(87,139,127)(83,43,31)(48,45,140)(61,44,60)

(144,88,105)(128,68,53)(59,92,142)

From above permutation formats of α and β, we see that 0 is mapped onto 72 through α and then β sent 72 to 19. This means that αβ maps 0 to 19. In this fashion, we can find the permutation form of αβ given below;

αβ=(0,19,47,116)(148,95,68,111)(1,∞,22,86)(147,21,119,94)(2,25,78,53)

(146,133,127,132)(3,98,42,142)(145,81,9,41)(4,23,56,125)(144,96,113,34)

(5,60,17,106)(143,128,137,102)(6,112,18,66)(141,121,63,135)(7,97,36,120)

(140,126,115,73)(8,65,43,54)(139,103,79,108)(10,72,85,38)(138,59,76,28)

(11,61,83,82)(136,75,16,52)(12,90,89,105)(134,87,100,13)(14,91,27,131)

(130,74,20,30)(15,118,129,107)(124,62,88,58)(24,61,104,77)(123,55,80,122)

(26,40,45,39)(117,49,50,92)(29,70,35,44)(114,84,110,48)(31,37,109,51)(101,71)

(33,64,93,69)(99,57,46,32)

**Fig. 2:**
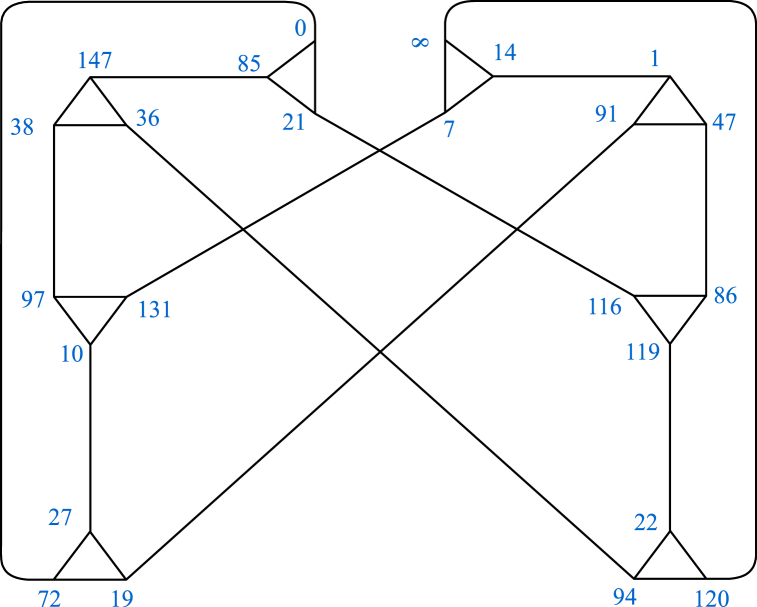
One of the patches $P_{i}$ of $D_{1}$.

**Fig. 3:**
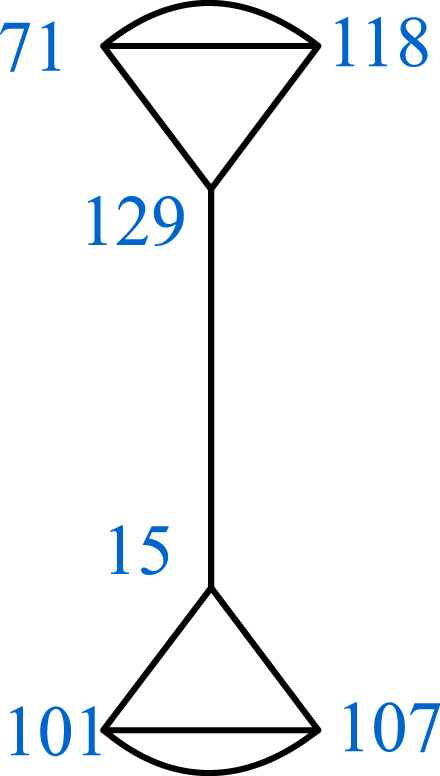
The patch $P_{7}$ of $D_{1}$.

It is easy to verify that the order of α,β and αβ is 2,3 and 4 respectively. Thus, the resulting coset graph $D_{1}$ represents the triangle group $\left\langle \alpha ,\beta =\alpha^{2}=\beta^{3}={\left(\alpha \beta \right)}^{4}=1\right\rangle$ over $F_{149}\text{.}$ It is a disconnected graph containing 7 patches. Out of these 7 patches of $D_{1}$, 6 patches $P_{i}$ (i=,2,3,4,5,6) are identical (See Fig. 2) to each other, except the labeling of vertices. Then 7th patch (See Fig. 3) is denoted by $P_{7}$.

### 3.2 Implementation of parametrization techniques to compose coset graph over $F_{107}$

According to parametrization method, we can draw coset graph for $\left\langle \alpha ,\beta =\alpha^{2}=\beta^{3}={\left(\alpha \beta \right)}^{n}=1\right\rangle$ over $F_{107}$ for all n dividing 106 or 108. Here we choose n=18. The polynomial associated with ${\left(\alpha \beta \right)}^{18}=1$ is ${f\left(t\right)=t}^{6}-{12t}^{5}+{54t}^{4}-112t^{3}+105t^{2}-36t+3$ which has roots 5,6,9,12,15 and 16. Let us use t=9 to solve Eqs 2.1, 2.5). In Eq (2.1), suppose Δ=1 to get r=3. Put k=1 in Eq (2.2) and obtain s=23. In equation (2.3), d=2 yields f=10. Next, solve Eqs 2.1, 2.5) to get a=20 and c=54. So, the required linear fractional transformation are $\alpha \left(x\right)=\frac{20x+54}{54x-20}$ and $\left(x\right)=\frac{2x+10}{10x-3}$ *.* The application of α and β on all elements of $F_{107}$ yield the following permutation representations.

α=(8,0)(84,1)(57,2)(3,43)(52,4)(34,5)(59,6)(92,7)(85,9)(10,33)(11,18)(86,12)(56,13)

(14,106)(15,53)(16,58)(96,17)(19,30)(20,83)(21,74)(22,64)(78,23)(24,67)(26,25)

(65,27)(76,28)(29,89)(88,31)(32,94)(105,35)(41,36)(37,77)(38,42)(39,44)(40,∞)

(45,82)(75,46)(47,70)(48,93)(49,99)(50,61)(51,98)(62,69)(91,63)(66,81)(68,101)

(71,102)(72,80)(73,95)(79,103)(87,90)(97,100)

β=(0,68,102)(43,11,∞)(17,15,1)(106,57,44)(2,26,96)(39,37,53)(58,3,64)(5,71,83)

(67,40,4)(80,8,99)(30,6,98)(12,89,82)(87,7,85)(73,61,91)(9,20,60)(41,16,95)

(55,10,104)(36,32,35)(38,13,66)(47,74,76)(50,14,94)(33,84,92)(22,18,19)(45,101,34)(77,21,69)(31,56,25)(23,29,105)(54,59,93)(63,48,24)(49,78,90)(75,27,86)(51,103,97)(65,28,52)(72,42,79)(62,46,81)(70,100,88)

By using permutation forms of α and β, the permutation representation of αβ can be obtained and given below;

αβ=(0,99,78,29,82,101,102,83,60,55,59,98,103,72,8,68,34,71)

(106,94,35,23,90,7,33,104,9,87,49,80,42,13,25,96,15,39)

(1,92,85,20,5,45,12,75,81,38,79,97,88,56,66,62,77,53)

(105,36,16,3,11,19,6,93,24,40,43,64,18,∞,4,65,86,89)

(2,44,37,21,76,52,67,63,73,41,32,50,91,48,54,10,84,17)

(100,51,30,22,58,95,61,14,57,26,31,70,74,69,46,27,28,47)

**Fig. 4:**
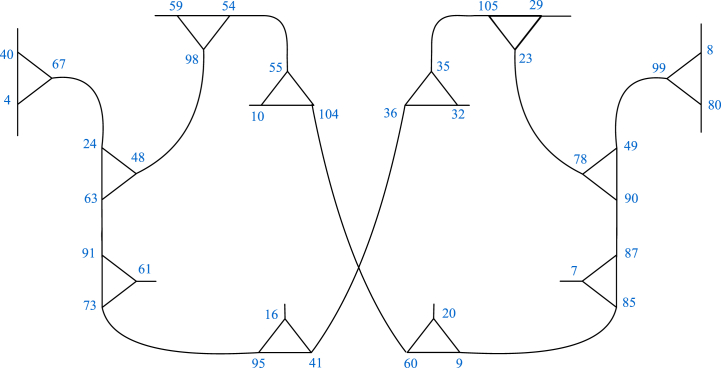
Some part of $D_{2}$.

From above permutation representations, it is easy to verify that the order of α,β and αβ is 2, 3 and 18 respectively. Thus, the resulting coset graph $D_{2}$ represents the triangle group $\left\langle \alpha ,\beta =\alpha^{2}=\beta^{3}={\left(\alpha \beta \right)}^{18}=1\right\rangle$ over $F_{107}\text{.}$ It is a connected graph and some part of it is shown in Fig. 4.

### 3.3 Arrangement and mixing of αβ cycles of $D_{1}$ and $D_{2}$

We use cycles of the permutations αβ of $D_{1}$ and $D_{2}$ in a certain way to break the initial sequence 0,1,2,..,255. The number of cycles in αβ for $D_{1}$ and $D_{2}$ are 38 and 6 respectively. First, we write them in a certain pattern. We call first cycle of $D_{1}\left(D_{2}\right)$ in which the least element of $F_{149}\;\left(F_{107}\right)$ that is 0 is present and denote this by $\lambda_{1}^{\left(0\right)}\left(\rho_{1}^{\left(0\right)}\right)$. The second cycle of $D_{1}\left(D_{2}\right)$ will be that cycle in which the largest element 148 (106) is lying and is denoted by $\lambda_{2}^{\left(148\right)}$ $\left(\rho_{2}^{\left(106\right)}\right)$. The third, fifth and seventh cycles of $D_{1}\left(D_{2}\right)$ are those cycles having available second, third and fourth least elements respectively. In the same way the fourth, sixth and eighth cycles of $D_{1}\left(D_{2}\right)$ will be those cycles containing available second, third and fourth largest elements respectively. This process continues till all the cycles get consumed. Therefore, the arrangements for all cycles of $D_{1}$ and $D_{2}$ are given below as arrangement 1 and arrangement 2, respectively:

#### 3.3.1 Arrangement 1

$$\lambda_{1}^{\left(0\right)},\lambda_{2}^{\left(148\right)},\lambda_{3}^{\left(1\right)},\lambda_{4}^{\left(147\right)},\lambda_{5}^{\left(2\right)},\lambda_{6}^{\left(146\right)},\lambda_{7}^{\left(3\right)},\lambda_{8}^{\left(145\right)},\lambda_{9}^{\left(4\right)},\lambda_{10}^{\left(144\right)},\lambda_{11}^{\left(5\right)},\lambda_{12}^{\left(143\right)},\lambda_{13}^{\left(6\right)},\lambda_{14}^{\left(141\right)},\lambda_{15}^{\left(7\right)},\lambda_{16}^{\left(140\right)},\lambda_{17}^{\left(8\right)},\lambda_{18}^{\left(139\right)},\lambda_{19}^{\left(10\right)},\lambda_{20}^{\left(138\right)},\lambda_{21}^{\left(11\right)},\lambda_{22}^{\left(136\right)},\lambda_{23}^{\left(12\right)},\lambda_{24}^{\left(134\right)},\lambda_{25}^{\left(14\right)},\lambda_{26}^{\left(130\right)},\lambda_{27}^{\left(15\right)},\lambda_{28}^{\left(124\right)},\lambda_{29}^{\left(24\right)},\lambda_{30}^{\left(123\right)},\lambda_{31}^{\left(26\right)},\lambda_{32}^{\left(117\right)},\lambda_{33}^{\left(29\right)},\lambda_{34}^{\left(114\right)},\lambda_{35}^{\left(31\right)},\lambda_{36}^{\left(101\right)},\lambda_{37}^{\left(33\right)},\lambda_{38}^{\left(99\right)}$$

#### 3.3.2 Arrangement 2

$$\rho_{1}^{\left(0\right)},\rho_{2}^{\left(106\right)},\rho_{3}^{\left(1\right)},\rho_{4}^{\left(105\right)},\rho_{5}^{\left(2\right)},\rho_{6}^{\left(100\right)}$$

**Fig. 5:**
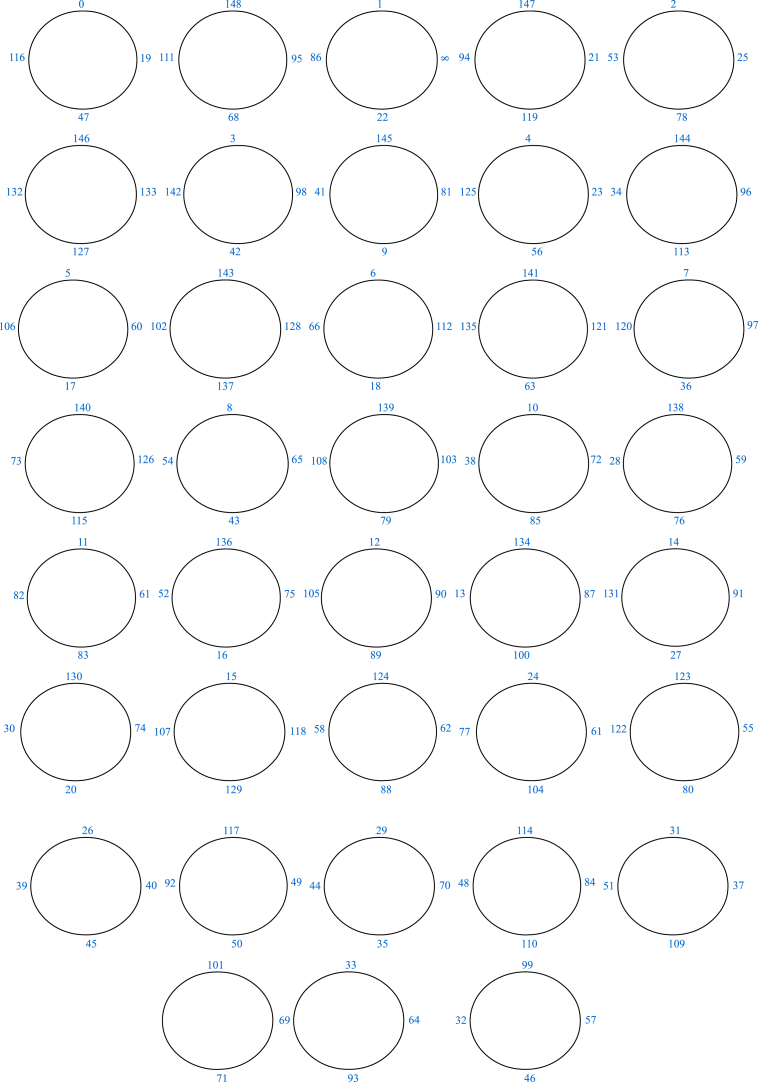
Graphical version of the arrangement 1.

**Fig. 6:**
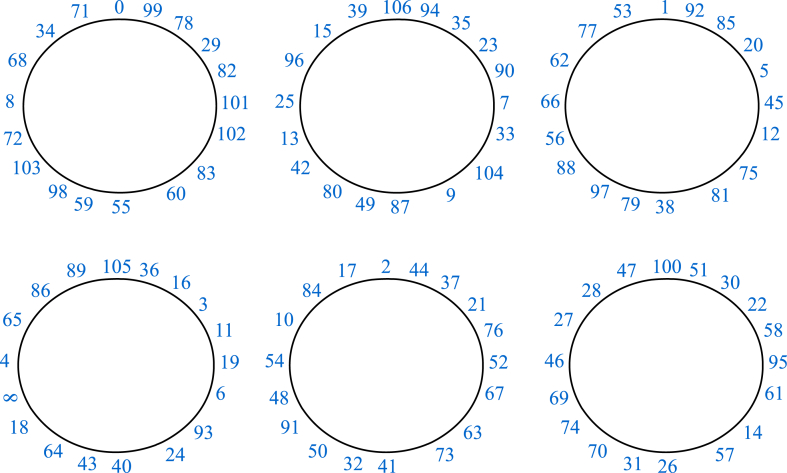
Graphical version of the arrangement 2.

The diagrammatic descriptions of the arrangements 1 and 2 are presented in Fig. 5, Fig. 6, respectively.

The aforementioned arrangements are combined in a ratio of 1:***6***:1:***6***:1:***7***:1:***7***:1:***6***:1:***6***. In this composition, each integer “1”, presented in regular font, represents a single cycle $\lambda_{i}^{\left(n\right)}$ of $D_{1}$ while bold integers “***6*** and ***7***” represent six and seven consecutive cycles $\rho_{j}^{\left(m\right)}$ of $D_{2}$ respectively. As a result, the final structure obtained is as follows;

#### 3.3.3 Arrangement 3

$$\rho_{1}^{\left(0\right)},\lambda_{1}^{\left(0\right)},\lambda_{2}^{\left(148\right)},\lambda_{3}^{\left(1\right)},\lambda_{4}^{\left(147\right)},\lambda_{5}^{\left(2\right)},\lambda_{6}^{\left(146\right)},\rho_{2}^{\left(106\right)},\lambda_{7}^{\left(3\right)},\lambda_{8}^{\left(145\right)},\lambda_{9}^{\left(4\right)},\lambda_{10}^{\left(144\right)},\lambda_{11}^{\left(5\right)},\lambda_{12}^{\left(143\right)}\rho_{3}^{\left(1\right)},\lambda_{13}^{\left(6\right)}\text{,}$$

$$\lambda_{14}^{\left(141\right)},\lambda_{15}^{\left(7\right)},\lambda_{16}^{\left(140\right)},\lambda_{17}^{\left(8\right)},\lambda_{18}^{\left(139\right)},\lambda_{19}^{\left(10\right)},\rho_{4}^{\left(105\right)},\lambda_{20}^{\left(138\right)},\lambda_{21}^{\left(11\right)},\lambda_{22}^{\left(136\right)},\lambda_{23}^{\left(12\right)},\lambda_{24}^{\left(134\right)},\lambda_{25}^{\left(14\right)},\lambda_{26}^{\left(130\right)},\rho_{5}^{\left(2\right)},\lambda_{27}^{\left(15\right)},\lambda_{28}^{\left(124\right)},\lambda_{29}^{\left(24\right)},\lambda_{30}^{\left(123\right)},\lambda_{31}^{\left(26\right)},\lambda_{32}^{\left(117\right)},\rho_{6}^{\left(100\right)},\lambda_{33}^{\left(29\right)},\lambda_{34}^{\left(114\right)},\lambda_{35}^{\left(31\right)},\lambda_{36}^{\left(107\right)},\lambda_{37}^{\left(33\right)},\lambda_{38}^{\left(99\right)}$$

Remark 3.1 Consider a cycle $\lambda_{i}^{\left(n\right)}$ of $D_{1}$. We use the term “pivot element” to refer to the element $n\in F_{149}$ in $\lambda_{i}^{\left(n\right)}$, which is either the least element in the cycle if it appears at an odd position in the arrangement 1, or the largest element of the cycle otherwise. Similarly, the element m is considered the pivot element in the cycle $\rho_{j}^{\left(m\right)}$ of $D_{2}$.

## 4 Proposed scheme

In this section, we describe the suggested scheme of S-box construction based on.

i. Coset graphs for two different groups over two different prime fields.
ii. A bijective function $t:F_{107}\cup F_{149}\to Z_{256}$.
iii. A newly defined operation of row and column vectors on square matrix

### 4.1 Step I

Arrange all elements of all cycles according to the arrangement 3 and compile them into a in a square matrix of order 16. It should be noted that each cycle must commence from the pivot element, and the infinities present in both graphs $D_{1}$ and $D_{2}$ must be disregarded. For instance, all 18 elements of $\rho_{1}^{\left(0\right)}$ should be positioned in the first row, followed by the first two elements of the second row in the same sequence as in $\rho_{1}^{\left(0\right)}$. Similarly, the next 23 elements (excluding $\infty \in \lambda_{3}^{\left(1\right)}$) of $\lambda_{1}^{\left(0\right)}\text{,}$ $\lambda_{2}^{\left(148\right)},\lambda_{3}^{\left(1\right)},\lambda_{4}^{\left(147\right)},\lambda_{5}^{\left(2\right)}$ and $\lambda_{6}^{\left(146\right)}$ fill the matrix up to the 9th element of the 3rd row, and so on. This process is repeated until all the cycles of $D_{1}$ and $D_{2}$ are accounted for, resulting in a 16×16 matrix as presented in Table 2.

**Table 2:** The outcome of Step I.

**0**	99	78	29	82	101	102	83	60	55	59	98	103	72	8	68
**34**	71	0	19	47	116	148	95	68	111	1	22	86	147	21	119
**94**	2	25	78	53	146	133	127	132	106	94	35	23	90	7	33
**104**	9	87	49	80	42	13	25	96	15	39	3	98	42	142	145
**81**	9	41	4	23	56	125	144	96	113	34	5	60	17	106	143
**128**	137	102	1	92	85	20	5	45	12	75	81	38	79	97	88
**56**	66	62	77	53	6	112	18	66	141	121	63	135	7	97	36
**120**	140	126	115	73	8	65	43	54	139	103	79	108	10	72	85
**38**	105	36	16	3	11	19	6	93	24	40	43	64	18	4	65
**86**	89	138	59	76	28	11	67	83	82	136	75	16	52	12	90
**89**	105	134	87	100	13	14	91	27	131	130	74	20	30	2	44
**37**	21	76	52	67	63	73	41	32	50	91	48	54	10	84	17
**15**	118	129	107	124	62	88	58	24	61	104	77	123	55	80	122
**26**	40	45	39	117	49	50	92	100	51	30	22	58	95	61	14
**57**	26	31	70	74	69	46	27	28	47	29	70	35	44	114	84
**110**	48	31	37	109	51	101	71	33	64	93	69	99	57	46	32

### 4.2 Step II

An S-box is a square matrix of order 16, comprising 256 elements with values ranging from 0 to 255, arranged randomly. Table 2 represents this matrix, which exhibits repeated entries and the same number of missing elements. To address this problem, a bijective function $h:F_{107}\cup F_{149}\to Z_{256}$ defined by

$$h\left(\alpha \right)=\left\{ \begin{array}{c} \alpha \;if\;\alpha \in F_{107}\;and\;is\;non-prime\\ \alpha +149\;if\;\alpha \in F_{107}\;and\;is\;prime\\ \alpha +107\;if\;\alpha \in F_{149}\;and\;\alpha -42\;is\;non-prime\\ \alpha -42\;if\;\alpha \in F_{149}\;and\;\alpha -42\;is\;prime \end{array}\right.$$

is employed on Table 2.

This way, a new matrix with 256 distinct entries is evolved (See Table 3). This matrix is referred to as the initial S-box, which has robust cryptographic properties (NL value = 101) to reinforce the block cipher. The subsequent step focuses on introducing more randomness and complexity to the S-box to further improve its security.

**Table 3:** The outcome of Step II (Initial S-box).

**0**	99	78	178	82	250	102	232	60	55	208	98	252	72	8	68
**34**	220	107	126	5	223	255	53	175	218	108	129	193	254	128	226
**201**	109	132	185	11	253	240	234	239	106	94	35	172	90	156	33
**104**	9	87	49	80	42	162	25	96	15	39	110	205	149	249	103
**188**	116	148	111	130	163	83	251	203	71	141	112	167	124	213	101
**235**	244	209	1	92	85	20	154	45	12	75	81	38	228	146	88
**56**	66	62	77	202	113	219	125	173	248	79	170	242	114	204	143
**227**	247	233	73	31	115	23	150	161	246	61	37	215	117	179	43
**145**	105	36	16	152	160	168	6	93	24	40	192	64	18	4	65
**86**	238	245	17	183	135	118	174	41	189	243	182	123	159	119	197
**47**	212	241	194	207	120	121	198	134	89	237	181	127	137	151	44
**186**	21	76	52	216	63	222	190	32	50	91	48	54	10	84	166
**122**	225	236	214	231	169	195	165	131	19	211	184	230	13	187	229
**133**	147	3	97	224	7	157	199	100	51	30	22	58	95	210	14
**57**	26	180	70	74	69	46	27	28	196	136	177	142	2	221	191
**217**	155	138	144	67	158	59	29	140	171	200	176	206	164	153	139

### 4.3 Step III

**Fig. 7:**
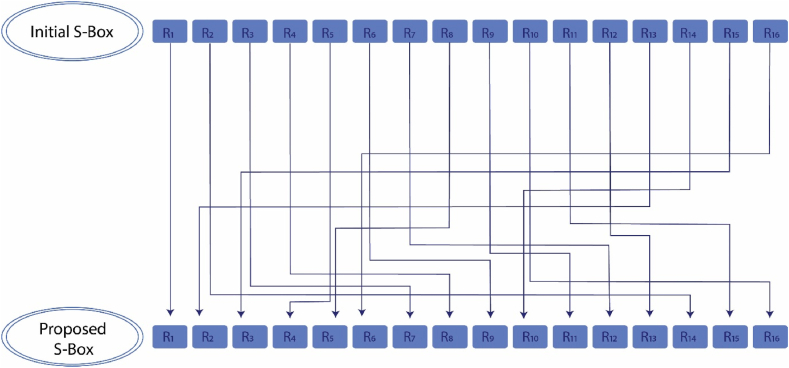
Graphic of the row vector's operation on the initial S-box.

**Fig. 8:**
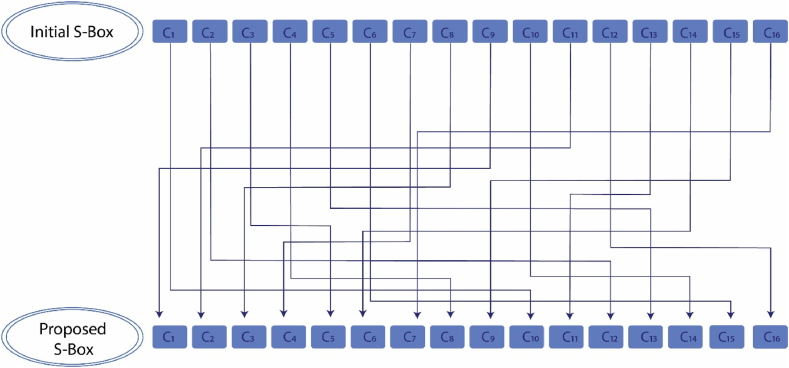
Graphic of the column vector's operation on the initial S-box.

The performance and quality of an S-box can be enhanced by operating (See Definition 2.1) row and column vectors R and C on it simultaneously. Here, we take R=[11478491251116151321036] and $C={\left[10\;12\;5\;8\;13\;15\;4\;3\;1\;14\;2\;16\;11\;6\;9\;7\right]}^{T}$. Then the operations of R and C on the initial S-box evolve the proposed S-box presented in Table 4. Fig. 7, Fig. 8 provide a graphical description of the operation of the row and column vectors on the initial S-box, respectively

**Table 4:** The proposed S-box.

**60**	208	232	102	78	72	68	178	8	0	252	99	82	55	250	98
**131**	211	165	195	236	13	229	214	187	122	230	225	231	19	169	184
**28**	136	27	46	180	2	191	70	221	57	142	26	74	196	69	177
**203**	141	251	83	148	124	101	111	213	188	167	116	130	71	163	112
**161**	61	150	23	233	117	43	73	179	227	215	247	31	246	115	37
**140**	200	29	59	138	164	139	144	153	217	206	155	67	171	158	176
**239**	94	234	240	132	90	33	185	156	201	172	109	11	106	253	35
**96**	39	25	162	87	149	103	49	249	104	205	9	80	15	42	110
**45**	75	154	20	209	228	88	1	146	235	38	244	92	12	85	81
**100**	30	199	157	3	95	14	97	210	133	58	147	224	51	7	22
**93**	40	6	168	36	18	65	16	4	145	64	105	152	24	160	192
**173**	79	125	219	62	114	143	77	204	56	242	66	202	248	113	170
**32**	91	190	222	76	10	166	52	84	186	54	21	216	50	63	48
**175**	108	53	255	107	254	226	126	128	34	193	220	5	218	223	129
**134**	237	198	121	241	137	44	194	151	47	127	212	207	89	120	181
**41**	243	174	118	245	159	197	17	119	86	123	238	183	189	135	182

**Fig. 9:**
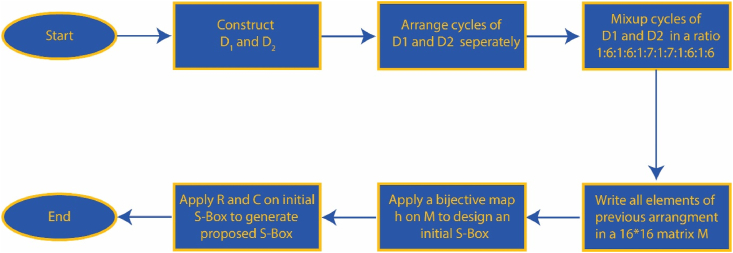
Block diagram describing the suggested S-box construction.

A block diagram has been presented in Fig. 9 to illustrate the construction of the suggested S-box design .

## 5 Experimental setup and analysis

This section details the experimental design and analysis aimed at assessing the efficacy of the proposed scheme [39]. Specifically, it covers the simulation platform used, the algebraic performance metrics utilized, a brief discussion of the algebraic analysis results, the Friedman test used to validate the superiority of the proposed model compared to other S-box models and limitations of the proposed scheme for S-box construction.

### 5.1 Simulation platform

The simulations were conducted using the computer language MATLAB. To minimize any potential bias in interpreting the data, the simulations were performed on a single computer equipped with an Intel Core i5 CPU operating at 3.2 GHz Quad-Core Processor and 16 GB RAM, running on the Windows 10 operating system. To ensure consistency in the experimental approach and control for any confounding variables, it is necessary to replicate the experiment using the same compiler and running it on a computer system with equivalent processing capability.

### 5.2 Performance metrics and analyses

In this era of 21st century when technology has advanced to new heights, the researchers have a significant difficulty, when it comes to safe communication. This section presents the performance evaluation of the suggested S-box under different state of the art performance metrics such as the nonlinearity test, strict avalanche criterion, bit independence criterion, differential uniformity, and linear approximation probability. We see that the outcome scores of our S-box obtained via these analyses are nearly equals to the ideal scores, demonstrating the effectiveness and capability of the proposed approach. The analyses applied on our S-box are as follows.

#### 5.2.1 Nonlinearity (NL)

Nonlinearity is a key factor to examine the robustness of an S-box. If an S-box maps input to output linearly, its resistance is very low [40]. A robust S-box nonlinearly maps input to output. Any S-box with a higher nonlinearity value guarantees more security against cryptanalytic attacks. In the case of Boolean function of the form $\theta :F_{2}^{n}⟶F_{2}$, The nonlinearity is computed as.

Nθ=2n−1−12[|Sθ(h)|h∈GF(2)nmax]

Note that, $S_{\theta }\left(h\right)=\sum_{g\in GF{\left(2\right)}^{n}}{\left(-1\right)}^{\theta \left(g\right)⊕g.h}$ represents the Walsh spectrum of θ(g). Table 5 indicates the nonlinearity values of all Boolean functions of the proposed S-box. The mean Non-linearity of our S-box is 107.75. A comparison of our S-box with some recently designed S-boxes in terms of NL score is given in Table 10.

**Table 5:** NL Values of all Boolean mappings involved in suggested S-box.

**Boolean mapping**	$\theta_{0}$	$\theta_{1}$	$\theta_{2}$	$\theta_{3}$	$\theta_{4}$	$\theta_{5}$	$\theta_{6}$	$\theta_{7}$	**Mean**
NL score	106	108	110	108	106	110	108	106	107.75

#### 5.2.2 Strict avalanche criteria (SAC)

SAC is effective technique to check the security of an S-box [48]. To meet this requirement, the input bit of any cryptosystem must change along with a 50% change in the output bits. The SAC performance of the S-box is evaluated by the dependency matrix. The perfect SAC score for the best cryptographic confusion is 0.5. Tables 6 shows the dependency matrix of SAC values obtain by proposed S-box. The mean SAC value of proposed S-box is 0.4983, which differs slightly from the ideal score. Thus, our S-box fulfills the SAC criterion.

**Table 6:** SAC values of constructed S-box.

0.4062	0.5	0.4844	0.4844	0.5469	0.4844	0.5156	0.5
0.4844	0.5	0.4844	0.4688	0.4531	0.4844	0.5	0.5625
0.4844	0.4688	0.5156	0.5469	0.4531	0.5	0.4688	0.4844
0.4375	0.4375	0.5156	0.4844	0.4844	0.4844	0.5469	0.4688
0.5156	0.5156	0.4688	0.5469	0.4375	0.5625	0.5	0.4531
0.4375	0.5	0.4844	0.4531	0.5156	0.5156	0.5469	0.5156
0.5625	0.4375	0.5469	0.4688	0.5938	0.5	0.5781	0.5156
0.5156	0.4688	0.5	0.5	0.4531	0.5156	0.5625	0.5625

#### 5.2.3 Bit independence criterion (BIC)

This test [48] is satisfied if the output bits operate independently, i.e. do not depend on each other. More specifically, no statistical dependencies or patterns should be present in the bits of the output vectors. It is intended to boost output bit autonomy for greater security. . Table 7, Table 8 depict the dependency matrices for BIC-nonlinearity and BIC-SAC respectively. The results show that the proposed S-box conforms to the required BIC standards.

**Table 7:** BIC outcomes for nonlinearity related to newly constructed S-box.

-	104	100	104	104	108	104	106
104	–	108	106	104	102	102	106
100	108	–	104	102	104	104	100
104	106	104	–	104	106	104	104
104	104	102	104	–	100	104	106
108	102	104	106	100	–	104	106
104	102	104	104	104	104	–	106
106	106	100	104	106	106	106	–

**Table 8:** BIC outcomes for SAC related to newly constructed S-box.

-	0.5098	0.4863	0.5352	0.5215	0.4805	0.4863	0.4688
0.5098	–	0.5039	0.5	0.5156	0.498	0.4961	0.502
0.4863	0.5039	–	0.5215	0.5117	0.5039	0.5156	0.5488
0.5352	0.5	0.5215	–	0.4844	0.5059	0.5156	0.4883
0.5215	0.5156	0.5117	0.4844	–	0.4941	0.4766	0.502
0.4805	0.498	0.5039	0.5059	0.4941	–	0.4883	0.5137
0.4863	0.4961	0.5156	0.5156	0.4766	0.4883	–	0.4453
0.4688	0.502	0.5488	0.4883	0.502	0.5137	0.4453	–

#### 5.2.4 Linear probability (LP)

Modern block ciphers are designed to create as much complexity among the bits as possible to protect the privacy of the information and to offer protection against various decryption techniques employed by the cryptanalysts. It is accomplished by S-Box. The lower the value of LP, the better the capability of S-box to withstand linear attacks. The LP value of an S-box is calculated by using the following formula [49].

LP=|#{w∈K:w.Γw=S(w).Γw′}2n−12|Γw,Γw′≠0max

where $K=\left\{0,1,.\;.\;.,2^{n}\right\}$ and Γw and $\Gamma w^{\prime }$ are the input and output masks respectively . The LP score of constructed S-box is 0.1328.

#### 5.2.5 Differential uniformity (DU)

The differential uniformity (DU) examines the capability of S-box to resist the differential cryptanalysis [50]. To compute DU, an input differential $\Delta \sigma_{i}$ is uniquely mapped to an output differential $\Delta \rho_{i}$, for all i. For a given S-box, its value can be calculated by using the following formula:

DU=#{σi∈Γ:S(σi)⨁S(σi+Δσi)=Δρi}Δσi≠0,Δρimax

It is necessary to design an S-box with smaller DU score to counter differential cryptanalysis attacks. The maximum DU score of our S-box is 10 (See Table 9), indicating its ability to counter differential attacks.

**Table 9:** DU scores of newly constructed S-box.

8	4	6	6	4	6	6	8	8	6	6	8	6	8	8	6
6	8	8	6	6	8	6	6	8	6	6	6	10	6	6	10
6	6	8	6	6	6	8	6	8	6	6	6	10	8	6	6
6	6	8	6	6	6	8	6	6	6	6	8	6	6	6	8
6	6	10	6	6	6	6	8	8	6	6	8	6	6	6	6
8	6	6	6	8	6	6	10	6	8	6	8	8	8	4	8
6	6	6	6	6	6	6	6	8	6	6	8	6	8	8	6
6	8	8	8	6	6	6	6	6	6	6	8	6	8	6	8
8	6	6	8	6	6	8	10	8	8	6	6	6	6	6	6
6	6	8	6	6	6	6	8	6	6	6	8	6	6	6	8
6	6	8	6	6	8	6	6	8	8	6	6	6	8	6	8
8	8	6	8	6	6	6	6	8	6	6	8	8	8	8	8
8	6	6	6	6	6	10	4	6	6	4	6	8	6	6	6
8	6	6	4	6	8	6	8	6	6	6	6	6	6	6	8
8	6	8	6	8	8	8	6	6	6	6	6	6	8	6	6
6	8	6	6	6	8	6	6	6	8	6	6	8	8	8	0

**Table 10:** Comparison of the various analyses between different S-boxes.

S-box	Nonlinearity Min Max Average			SAC	BIC-SAC	BIC-NL	DU	LP
Proposed S-box	106	110	107.75	0.4983	0.5007	104.14	10	0.1328
Ref [41]	106	108	106.25	0.5112	0.4975	103.93	12	0.1484
Ref [42]	106	108	107	0.4949	0.5019	102.29	12	0.141
Ref [43]	106	110	108.5	0.4995	0.5011	103.85	10	0.109
Ref [44]	108	110	109.75	0.5042	0.4987	110.6	6	0.0859
Ref [45]	102	110	106.5	0.4943	0.5019	103.35	12	0.1468
Ref [46]	104	108	105.5	0.5065	0.5031	103.57	10	0.1328
Ref [47]	104	110	107	0.4993	0,5050	103.29	10	0.1328

### 5.3 Discussion of performance results

Based on the comparative analysis conducted in this study, it was observed that the suggested technique for designing S-boxes resulted in better cryptographic features compared to many other recently designed S-boxes that utilized optimization, chaos and algebraic techniques. Table 10 presents a comparison of the different S-boxes, which clearly demonstrates the superiority of our proposed approach.

1. One of the important factors that contribute to the robustness of an S-box is its ability to resist linear attacks. This is typically measured by the Non-Linear (NL) score of the S-box. The NL value of an S-box is a measure of its degree of complexity and confusion-making ability, which are desirable properties for resisting linear cryptanalysis. As shown in Table 10, the suggested S-box has an average NL of 107.75, which is higher than the average NL scores of the S-boxes designed in Refs. [41,42,[45], [46], [47]]. This implies that our S-box has a higher degree of complexity and is more capable of creating confusion, thereby making it more resistant to linear cryptanalysis.
2. Strict avalanche criterion (SAC) is an important property of an S-box that measures its ability to resist cryptographic attacks. In the context of S-box design, achieving a SAC score as close to the optimal value (0.50) as possible is a primary objective. In Table 10, our S-box was found to have a SAC score of 0.4983, which is superior to the majority of recently constructed S-boxes based on optimization, chaos, and algebraic techniques, including [42,[44], [45], [46]]. This score is also extremely close to the ideal SAC value of 0.50. The close proximity of our S-box's SAC score to the ideal value indicates that it satisfies the requirements of strict avalanche criterion well. This property is important for ensuring the security of cryptographic systems. By exhibiting a strong SAC score, the S-box is able to provide greater resistance against various cryptographic attacks, including differential and linear cryptanalysis.
3. The obtained readings of BIC-NL and BIC-SAC from the proposed S-box are highly encouraging, as they outperform a significant number of S-boxes presented in Table 10. This outcome suggests that the suggested S-box has better properties in terms of BIC-NL and BIC-SAC compared to a considerable number of S-boxes designed through different approaches.
4. A potent S-box has a smaller DU value. As seen in Table 10, the DU score of the suggested S-box is less than or equal to the DU scores of S-boxes developed in Refs. [[41], [42], [43],[45], [46], [47]], thereby showing the acceptable robustness to differential cryptanalysis.
5. A smaller LP score makes an S-box more resistant to linear cryptanalysis. The LP score of our S-box is 0.1328, which is slightly higher than AES but lower than or equal to the LP values of many other recent S-boxes investigated in studies such as [41,42,[45], [46], [47]].

### 5.4 Friedman test

The Friedman test is a non-parametric statistical test that is used to analyze differences between two or more related groups [51,52]. It is similar to the repeated measures ANOVA test, but it does not require the assumption of normality or equal variances. The results of applying the Friedman test to the data are as follows. For the mentioned data, the degree of freedom is 7, default alpha is 0.05, and critical chi-square is 14.067. The applied the Friedman test which gives a p-value of *p* = *0.002897862191825* is considerably lower than the alpha value of 0.05 and the obtained chi-square is 21.67 which pretty higher than critical score. Thereby, it rejects the null hypothesis of equal performance of all S-boxes. The comparison made in Table 10 clearly indicates the better cryptographic performance of the proposed S-box over the existing ones.

### 5.5 Limitations of the proposed scheme for S-box construction

The construction of S-boxes has been a subject of intense research due to its significance in modern cryptography. Previous graphical methods for constructing S-boxes relied on a single graph to generate the substitution table. However, the present approach utilizes a combination of two graphs, which enhances the strength and security of the generated S-boxes. Our evaluation of the performance of the S-boxes generated through this approach has yielded promising results for almost all metrics. The major limitation of the proposed scheme is that we cannot obtain a bijective S-box directly from the combination of a pair of graphs. Although a random sequence of 256 elements was obtained, it contains numerous duplicated entries and an equal number of missing entries. However, this flaw is addressed in our study by introducing a bijective function that maps the sequence to $Z_{256}$. We ensure that the applied bijective function must retain the sequence pattern to the greatest degree possible, solving the issues of repetition and missing values at the same time. Although, this approach successfully resolves the issue of obtaining a bijective S-box, there are potentially other approaches that could also be used to address this limitation

## 6 Image encrption applications

**Fig. 10:**
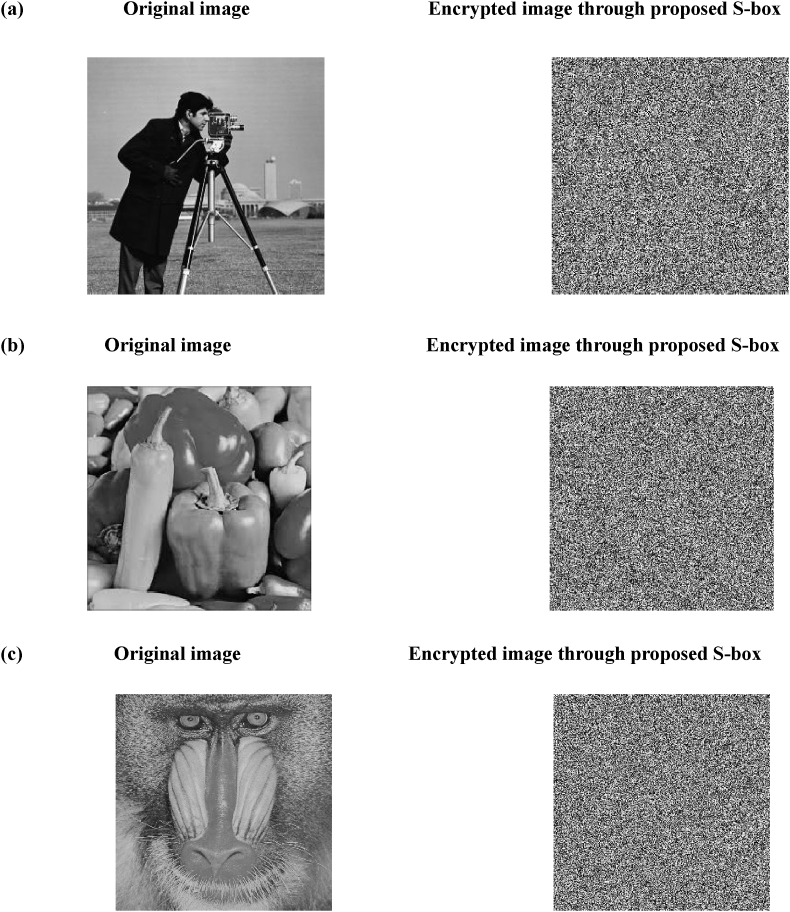
Original image and the encrypted images using two rounds of encryption a) Cameraman, b) Pepper and c) Baboon.

In order to determine the suitability of an S-box for encryption, its statistical competence is typically evaluated using the widely recognized majority logic criterion (MLC) [53]. Various statistical and analytical methods have been proposed in the literature to assess the S-box's confusion generating capacity. Due to the image distortion caused by the encryption process, it is important to understand the effects of statistical characteristics. The MLC consists of several tests, including entropy, contrast, homogeneity, correlation, and energy. We demonstrate the applicability of a proposed substitution-box for multimedia security and image encryption by using it to encrypt digital images. The proposed substitution-box is evaluated using the MLC and found to exhibit satisfactory statistical competence, indicating its potential for use in encryption applications. In the present study, three standard 256 × 256 sized grayscale images, namely Pepper, Cameraman, and Baboon, sourced from the USC-SIPI image database [54], and were selected for image processing. The proposed encryption procedure consists of two rounds of substitution utilizing an S-box. In the first round, the substitution process is executed in the forward direction, i.e., from the first pixel to the last pixel of the image. Wherein the image pixels are substituted with the help of proposed S-box in random fashion using cipher-block-chaining mode of encryption operations. In second round, the obtained forwardly substituted image obtained is passed through another substitution process but in the backward direction, i.e., from the last pixel to the first pixel using randomly picking S-box elements for substitution in cipher-block chaining mode. All experimental and simulation activities were performed using the MATLAB. The pseudo-code of the encryption scheme using proposed S-box S is provided in Table 11. Fig. 10 $\left(a-c\right)$ illustrates the original images and their encrypted versions obtained using the proposed S-box. The encrypted images exhibit a high degree of dissimilarity and indistinguishability compared to their respective plain-images. This is attributed to the absence of any discernible patterns that may reveal even the slightest information of the original image data. Consequently, the encrypted images display a considerable amount of visual distortion.

**Table 11:** The Pseudo code of the algorithm.

//Read S-box S and the original image PP S = Read (‘sbox.txt’) PP = Read (‘org_image.png’) [M, N] = size (PP) Len = M*N //Perform forward substitution on the image PP using S CP = sub_byte_f (PP, S) as below CF = 67; PP = reshape (PP, 1, Len) for k = 1:1:Len val = bitxor (PP(k), CF); t = mod (k, 256) + 1; CP(k) = bitxor (val, S(t)); CF=CP(k); end //Perform backward substitution on the image CP using S CC = sub_byte_b (CP, S)as below CB = 235; CP = wrev (CP);//reverse the 1D array CP for k = 1:1:Len val = bitxor (CP(k), CB); t = mod (k, 256) + 1; CC(k) = bitxor (val, S(t)); CB=C(k); end Perform encryption analysis MLC test on encrypted image CC.

### 6.1 Entropy

Entropy analysis is a common approach used to quantify the level of randomness present in encrypted images. Mathematically, entropy can be expressed as

$$H=-\sum_{j=0}^{n-1}p\left(x_{j}\right)\times \log_{2}\;p\left(x_{j}\right)$$

where H is the entropy value, $p\left(x_{j}\right)$ is the probability of occurrence of pixel intensity $x_{j}$, and the summation is taken over all possible pixel intensities. The entropy value ranges from 0 to the maximum entropy value for the image, which is dependent on the number of intensity levels present in the image. A higher entropy value indicates a higher level of randomness in the image.

Table 12 displays the entropy values of both the original and encrypted images. These findings indicate that in the image encryption process utilizing the suggested S-box, the degree of information leakage is negligible. This is substantiated by the considerably high entropy values exhibited by the encrypted images.

**Table 12:** MLC outcomes for the proposed S-box.

Images	Entropy	Contrast	Correlation	Energy	Homogeneity
***Cameraman Image***					
Before encryption	7.1025	0.4785	0.9292	0.1679	0.8964
After encryption	7.9836	8.6666	- 0.0019	0.0171	0.4115
***Pepper Image***					
Before encryption	7.5498	0.2668	0.9365	0.1477	0.9191
After encryption	7.9842	8.5788	−0.0120	0.0173	0.4052
***Baboon Image***					
Before encryption	7.1273	0.7179	0.6782	0.1025	0.7669
After encryption	7.9806	8.2341	−0.0060	0.0181	0.4102

### 6.2 Contrast

During the process of image processing, adjustments are made to ensure optimal viewing conditions for contrast and brightness. Contrast refers to the disparity in brightness among objects. The non-linear substitution of the S-box in the encryption process results in a direct correlation between contrast and image randomness. A uniform plain-image will possess a contrast value of zero. In most cases, an image's contrast is measured through the following means:

$$C=\sum_{j,K=0}^{m-1,n-1}p\left(j,k\right){\left|k-j\right|}^{2}$$

Here, p(j,k) denote the position of pixels in gray level co-occurrence matrices. The contrast values of three distinct sets of images are presented in Table 12. The encrypted images exhibit notably high contrast scores in comparison to the original images. This disparity indicates that the proposed encryption process has minimal information leakage.

### 6.3 Correlation

The correlation test is a commonly used technique to quantify the similarity between a plain image and its encrypted version. It involves comparing the pixel values of the original image with those of the encrypted image. The correlation coefficient is a measure of how closely the pixel values of neighboring pixels in the two images are related. A high correlation coefficient indicates that the two images are highly similar, while a low correlation coefficient indicates that the images differ significantly. A lower correlation value between the host and encrypted image implies that more distortion has been introduced by the encryption process.

### 6.4 Energy

In energy analysis, the calculation of the sum of squared gray level co-occurrence members is utilized. In the gray level co-occurrence matrix, pixels with high values occur in specific regions of a plain image, which results in a higher energy value. However, in an encrypted image, the energy values are more evenly distributed, leading to a smaller overall energy value compared to the original image. The energy analysis performed under maximum likelihood classification (MLC) is represented by a mathematical expression

$$E=\sum_{j,k}p{\left(j,k\right)}^{2}=$$

Table 12 displays the energy values of three plain and encrypted images. The significantly smaller energy values obtained for encrypted images indicate a positive encryption effect in comparison to the plain-images.

### 6.5 Homogeneity

Homogeneity is a measure of the closeness of the distribution of elements in the diagonal of the gray level co-occurrence matrix and the gray level co-occurrence itself, and it is evaluated through a mathematical procedure. The range of homogeneity values is between 0 and 1, and its magnitude is primarily influenced by the elements present in the diagonal of the gray level co-occurrence matrix. A lower homogeneity value in encryption indicates a stronger encryption algorithm. The calculation of homogeneity in digital image content is determined using the following formula;

$$H=\sum_{j,k}\frac{p\left(j,k\right)}{1+\left|k-j\right|}$$

The homogeneity scores computed for each of the three plain and encrypted images have been documented in Table 12. The results demonstrate that the homogeneity values for the encrypted image are notably lower than those of the original image, reaffirming the significant impact of the encryption process.

## 7 Conclusion

This paper presents a novel approach for developing reliable S-boxes. The approach is based on coset graphs for $F_{107}\cup \infty$ and $F_{149}\cup \infty$ along with a newly defined operation of row and column vectors R and C on a square matrix. An initial S-box is generated by defining a precise method for selecting the vertices from both graphs and setting them at specific positions within the S-box matrix. Then the operation of R and C on the initial S-box evolves our S-box. The constructed S-box is examined for cryptographic strength using some performance evaluation metrics. In comparison to various contemporary S-boxes present in the literature, the performance outcomes of the proposed S-box are found to be superior. The S-box is used to encrypt the digital images, and the outcomes of the majority logic criteria demonstrated good encryption quality in the encrypted content. Thus, the application of the proposed S-box in the field of image encryption suggests its suitability for ensuring data security during transmission over an insecure channel.

It is not always possible to construct a uniform distribution among characteristics of an S-box that can survive a broad variety of cryptanalysis assaults. This is the most difficult aspect of constructing an S-box. The majority of S-boxes documented in academic literature provide effective cryptographic security against certain assaults, but inadequate protection against others. The suggested S-box is suitable for use in image encryption applications and has strong algebraic properties for resisting linear and differential assaults.

In terms of practical applications, the proposed S-box could be integrated into existing encryption protocols to enhance their security and ensure the confidentiality of sensitive data during transmission over insecure channels. The direction of future research in cryptography should focus on developing more robust and secure encryption techniques that can withstand advanced cryptanalysis attacks. The use of innovative approaches, such as the coset graphs and *R* and *C* operations proposed in this paper, could lead to the development of more effective encryption methods and contribute to the advancement of the field of cryptography. Furthermore, the use of this S-box in image encryption applications suggests that it could be used in other multimedia encryption scenarios, such as video or audio encryption.

## Author contribution statement

Abdul Razaq: Conceived and designed the experiments; Performed the experiments; Contributed reagents, materials, analysis tools or data; Wrote the paper.

Ghaliah Alhamzi: Contributed reagents, materials, analysis tools or data.

Sajida Abbas: Conceived and designed the experiments; Performed the experiments.

Musheer Ahmad: Analyzed and interpreted the data; Wrote the paper.

Asima Razzaque: Analyzed and interpreted the data; Contributed reagents, materials, analysis tools or data; Wrote the paper.

## Data availability statement

No data was used for the research described in the article.

## Additional information

No additional information is available for this paper.

## Declaration of competing interest

The authors declare no competing interests.
